# Mechanisms for Orofacial Pain: Roles of Immunomodulation, Metabolic Reprogramming, Oxidative Stress and Epigenetic Regulation

**DOI:** 10.3390/biomedicines13020434

**Published:** 2025-02-11

**Authors:** Saniyya Khan, Feng Tao

**Affiliations:** Department of Biomedical Sciences, Texas A&M University College of Dentistry, 3302 Gaston Ave., Dallas, TX 75246, USA; saniyyakhan@gmail.com

**Keywords:** orofacial pain, immunomodulation, metabolic reprogramming, oxidative stress, epigenetic regulation

## Abstract

**Background and Objectives**: Orofacial pain corresponds to pain sensitization originating from the facial and oral regions, often accompanied by diagnostic complexity due to a multitude of contributory factors, leading to significant patient distress and impairment. Here, we have reviewed current mechanistic pathways and biochemical aspects of complex orofacial pain pathology, highlighting recent advancements in understanding its multifactorial regulation and signaling and thus providing a holistic approach to challenging it. **Materials and Methods**: Studies were identified from an online search of the PubMed database without any search time range. **Results**: We have discussed neuron–glia interactions and glial cell activation in terms of immunomodulatory effects, metabolism reprogramming effects and epigenetic modulatory effects, in response to orofacial pain sensitization comprising different originating factors. We have highlighted the fundamental role of oxidative stress affecting significant cellular pathways as well as cellular machinery, which renders pain pathology intricate and multidimensional. Emerging research on the epigenetic modulation of pain regulatory genes in response to molecular and cellular environmental factors is also discussed, alongside updates on novel diagnostic and treatment approaches. **Conclusions**: This review deliberates the integrative perspectives and implications of modulation in the immune system, glucose metabolism, lipid metabolism and redox homeostasis accompanied by mitochondrial dysfunction as well as epigenetic regulation accommodating the effect of dysregulated non-coding RNAs for an interdisciplinary understanding of pain pathology at the molecular level, aiming to improve patient outcomes with precise diagnosis offering improved pain management and treatment.

## 1. Introduction

Orofacial pain is a clinical condition encompassing painful sensations originating from oral and craniofacial regions, and it represents one of the most rampant pain conditions [1]. Classically, it is characterized as odontogenic, comprising pathologies like dental caries, tooth decay, pulpitis and dentine hypersensitivity; however, any inflammatory, nociceptive, stress or tissue injury stimuli in oral and craniofacial regions contribute to its pathogenesis [2].

Owing to the complexity of the facial region, orofacial pain can be attributed to many sources, rendering its management complicated [3], and also subjecting its classification to being challenged with a high risk of misdiagnosis and mismanagement. The International Classification of Orofacial Pain (ICOP) provides the most accepted and widely recognized classification of orofacial pain. Broadly, it comprises six chapters detailing pain in dentoalveolar and anatomically related tissues, muscle pain, temporomandibular joint (TMJ) pain, neuropathic pain affecting cranial nerves, pain resembling primary headaches, and idiopathic pain [4].

Clinical conditions in the TMJ are defined as TMJ disorders (TMDs). TMDs are more frequent in women compared to men and are related to rampant cases of orofacial pain, comprising jaw ache, earache, toothache, facial pain, headache or facial pressure. These pain conditions are subcategorized into primary and secondary. Classically, the pain conditions worsen during jaw movement or palpation. Also, any myofascial pain sensation referring to the dentialveolar region is categorized under TMDs [4]. TMD cases are identified using magnetic resonance imaging, employed as a standard diagnostic tool [2]. Technically, TMDs are manifestations of impaired TMJ biomechanics resulting in disc displacement, internal disarrangement or osteoarthritic changes. Their treatment usually involves the use of analgesics, nonsteroidal anti-inflammatory drugs (NSAIDs), local anesthetics, oral and injectable corticosteroids, sodium hyaluronate injections, muscle relaxants, botulinum toxin injections and antidepressants [3].

Pain perception and transmission involve the stimulation of neurons as well as immune cells. An understanding of orofacial pain pathophysiology, i.e., origin, persistence, enhancement or alleviation, can support a better diagnosis and prognosis of orofacial pain leading to an appropriate pharmacological treatment as well as management plans. An orofacial pain signal is transmitted from the primary afferent neurons or pain-sensing neurons (nociceptors) to the central nervous system (CNS) via trigeminal ganglia (TG). During this, the associated non-neuronal immune cells are activated and supplement the pain signaling by contributing several immunomodulatory factors such as cytokines and neurotrophic and tumor necrosis factors. In non-nervous tissues, non-neuronal immune cells (e.g., macrophages) are involved in peripheral sensitization, while in the central nervous system, glial cells (e.g., microglia and astrocytes) contribute to central sensitization [5]. It is at this stage that these neuronal cells undergo several physiological changes in terms of altered cellular metabolism directing mitochondrial dysfunction [6,7,8]. In addition to this, a genome-wide association study (GWAS) on pain-specific genes has demonstrated that many pain-related genes exhibit altered expression at both the mRNA and protein levels, influencing the pathogenesis and persistence of chronic pain concurrently [9].

With the advancement in understanding pain pathways at a molecular and cellular level, it is now evident that pain signaling involves an interplay of immunomodulation, metabolic reprogramming and epigenetic modifications in the physiological systems of nerve and immune cells [1,3,5,7,9]. Here, in this review, we present a thorough insight into the disturbed homeostasis of neural cells and have signified the importance of understanding of pathophysiology of orofacial pain at the molecular level. Such comprehensive understanding can pave the way for developing novel strategies and treatment approaches resulting in better pain management.

We conducted comprehensive searches of the literature focusing on orofacial pain and its mechanisms in PubMed and Google Scholar. The following keywords were used for selecting the cited studies: immunoregulation of orofacial pain, oxidative stress in orofacial pain, redox homeostasis in orofacial pain, reactive radicals in orofacial pain, mitochondrial dysfunction in orofacial pain, epigenetic regulation of orofacial pain, non-coding RNAs in orofacial pain, and clinical studies of orofacial pain. The publications not available in English were excluded. We prioritized those studies published within the last ten years.

## 2. Glial Cell Activation and Orofacial Pain Chronicity

**Figure 1:**
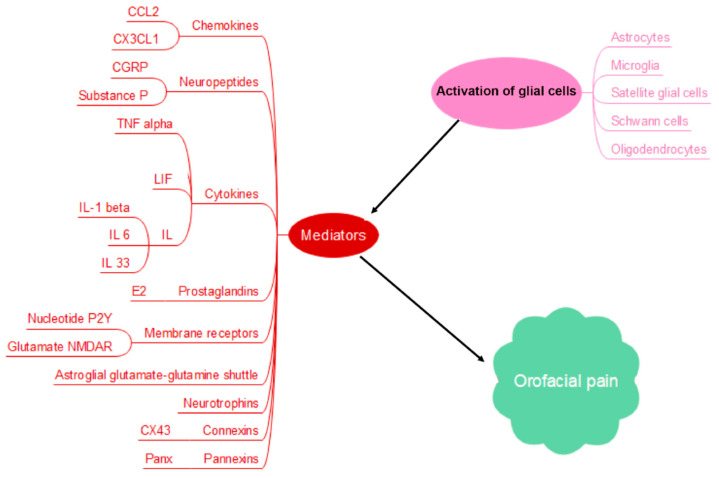
Glial cell activation and immunomodulation of orofacial pain through various mediators, including chemokines like CCL2 and CX3CL1, neuropeptides like calcitonin gene-related peptide (CGRP) and Substance P, cytokines like TNFα, neurotrophins like BDNF and NGF, leukemia inhibitory factor (LIF), interleukins (IL-1β, IL-6, IL33), receptors like nucleotide P2Y receptors and glutamate N-methyl-d-aspartate receptor (NMDAR), prostaglandins like E2, astroglial glutamate–glutamine shuttle, gap junctions connexins like CX43, and pannexins like Panx.

Immune cells impart a homeostatic environment in the brain as they continuously patrol the brain parenchyma, acting as sensors for any physiological alteration [10,11]. They execute a profound role in pain signaling as they interact with neurons while directly influencing their functional capability of excitability [12,13,14,15]. Generally, macrophages as well as glial cells exhibit functional and structural heterogeneity, potentiating pain signaling through various mediators such as chemokines, neurotrophins, neuropeptides, prostaglandins and cytokines which bind to their respective receptors expressed on nociceptive primary afferent neurons. Factors like C-C motif chemokine ligand 2 (CCL2), C-X3-C motif chemokine ligand 1 (CX3CL1), brain-derived growth factor (BDFN), tumor necrosis factor alpha (TNFα), nerve growth factor (NGF), leukemia inhibitory factor (LIF), interleukin 1 beta (IL-1β) and interleukin 6 (IL-6) all have been characterized with distinct roles in pain pathophysiology [12,15,16,17]. Glial cells like astrocytes, microglia, oligodendrocytes, satellite glial cells (SGCs) and Schwann cells contribute to immunomodulation of orofacial pain [18], including both the development and chronic progression of orofacial pain. It has been reported that SGCs derive neuron–glia interactions as well as signaling in TG generating peripheral sensitization; on the other hand, microglia and astrocytes in the CNS contribute to central sensitization in spinal trigeminal nucleus caudalis (Sp5C) [19]. Figure 1 shows a brief outline of chief contributors in immunomodulatory system of orofacial pain pathophysiology.

The increase in orofacial pain in patients receiving cancer therapy has also been attributed to the vasodilation and vasoconstriction effect of astrocytes that release mediators into the vascular system [20]. The role of activated astrocytes and microglia in craniofacial cancers and tongue cancer has also been documented [21,22,23]. The pain-augmenting role of activated Schwann cells in oral carcinogenesis is mediated by the release of TNFα. The Schwann cells reciprocate with cancer cells and increase adenosine production with enhanced secretion of IL-6, TNFα and NGF, which ultimately have an augmenting effect on cancer proliferation, migration and invasion [24,25].

It has been reported that migraine-associated genes carry signal transduction and protein modification in astrocytes and oligodendrocytes [26]. In temporomandibular joint (TMJ) inflammation, activation of SGCs has been reported in the TG [27], and the activation of microglia as well as astrocytes has been reported in the Sp5C [18]. The activated SGCs are facilitated by the upregulation of purinergic G-protein-coupled nucleotide P2Y receptors (P2Y1 and P2Y2) [28]. They exhibit increased intercoupling and formation of gap junctions which ultimately aid in signal modulation [29,30]. They also release several pro-inflammatory cytokines and prostaglandins such as IL1β and PGE2 facilitating neuronal hyperexcitability through overexpression of voltage-dependent sodium channel Nav1.7 in TG neurons [31]. In a TMJ pain mouse model, we demonstrate the crucial role of trigeminal TNFα, which is regulated by a DNA methylation-mediated epigenetic mechanism [32]. New findings have widened the role of oligodendrocytes in neuropathic pain, in which oligodendroglia exhibit an enhancement in the production of cytokines, such as interleukin 33 (IL-33), leading to pain exacerbation [33]. Thus, all these studies project a strong foothold of immunomodulatory action of different glial cells at various stages of pain sensitization, leading to substantial alterations in pain pathophysiology.

## 3. Metabolic Reprogramming and Orofacial Pain

The severity and maintenance of orofacial pain have been related to the observed metabolic changes in neurons and glial cells. The shift in cellular energy metabolism is central to pain physiology and is widely being investigated for a profound understanding of pain pathology [34]. Glucose is a carbohydrate biomolecule that serves as a primary energy provider of the nervous system. It is taken up by the brain cells, crossing the blood–brain barrier via facilitated diffusion through sodium-independent transporters known as GLUT1-6 and GLUT8, as well as being co-transported along with sodium ion via SGLT1 [35]. It is sourced either through dietary supplementation or through glycogenolysis as well as gluconeogenesis. The glucose molecule starts the energy pathway by undergoing glycolysis followed by the tricarboxylic acid (TCA) cycle and oxidative phosphorylation ultimately producing ATP, the energy currency of cells. The ATP requirement of nerve cells depends upon the condition of the cells. For more neuronal activity or immune cell activation, more ATP is required [36]. In addition to ATP generation, glucose contributes to the synthesis of various neurotransmitters/neuromodulators, transmembrane ionic concentration gradients, neuronal plasticity, redox status, learning and memory [37].

Under normal conditions, pyruvate is produced as an end product of glycolysis that enters the TCA cycle, generating 32 ATP via oxidative phosphorylation; however, in hypoxic conditions, pyruvates are converted to lactate via anaerobic respiration, generating 2 ATP molecules only [38]. The anaerobic route yields less ATP at a higher rate as compared to oxidative phosphorylation which is more efficient but has a slower rate. Immune cells derive their ATP primarily from oxidative phosphorylation at the resting state; however, upon activation, their ATP supply shifts to the anaerobic route to facilitate faster energy supply. This leads to glycometabolism reprogramming characterized by impaired oxidative phosphorylation and an enhanced glycolytic rate. It has been established that upon nerve injury, the neurons and supporting immune cells undergo glycometabolism reprogramming affecting pain sensitization, development and transmission via altered synaptic plasticity and neuronal excitability [8]. Glucose metabolites have a direct role in pain mechanisms as they can alter the expression of pain-related genes via substrate-level gene modification. A perturbed oxidative phosphorylation results in an imbalanced NAD^+^/NADH ratio which directly affects the acetylation–deacetylation cycle of histones, thereby triggering epigenetic regulation of pain-specific genes [8,39].

Altered levels of several metabolic intermediates such as succinate, lactate, citrate, α-ketoglutarate (α-KG), 2-hydroxyglutarate (2-HG), S-adenosylmethionine (SAM) and fructose-1,6-bisphosphate (FBP) can influence pain sensitization and provide mechanistic insights into their respective roles at the molecular level [8]. Succinate plays a crucial role in inflammatory kinetics. Its cellular and extracellular accumulation leads to hypoxia-inducible factor-1α stabilization, thereby enhancing inflammation by inhibiting prolyl hydroxylases, and it can also signal and modulate immune cell activity using succinate receptor 1 on nerve cells [40]. Extensive studies have shown that lactate acts as an excellent sensor and signaling molecule imparting its effect on various physio-pathological processes of brain metabolism as well as synaptic plasticity [41,42]. Its anti-inflammatory effect projects it as an emerging therapeutic target for inflammatory orofacial pain. It regulates the secretion of IL-1β, IL-10, NF-κB and IL-8 through various pathways [43,44,45]. In the brain, lactate serves as the primary fuel for neurons or glial cells. The excitatory synaptic activity runs primarily on lactate transporting neuron–glia neurometabolic coupling known as the astrocyte-to-neuron lactate shuttle (ANLS) [46]. Thus, fluctuations in lactate levels can directly affect the functioning of neurons involved in pain sensation. Recent findings suggest the lactate-mediated neuro-glia crosstalk as an indicator of pain sensitization. A reduction in lactate transport through this shuttle can attenuate nociceptive, inflammatory and pathological pain. It has been reported recently that a reduction in lactate shuttling via ANLS attenuates opioid-induced hyperalgesia as well as central sensitization [47]. On the other hand, the transfer of lactate via ANLS can persistently sensitize neurons, resulting in chronic neuropathic pain [48,49]. The modulating effects of citric acid, α-KG, 2-HG and SAM on macrophage activity and T-cell proliferation have been well documented, implicating their respective roles in pain sensitization [8]. Fructose 1,6-bisphosphate not only holds anti-inflammatory potential by inhibiting cytokine and adenosine production but also has an antinociceptive effect on hyperalgesia [50].

Generally, cellular lipids along with their metabolites act as endogenous ligands in several pathways, and their association with pain signaling can produce pain-augmenting effects. Both glial cells and neurons can be the source of these molecules in response to either inflammation or oxidative stress. Oxides and epoxides of essential fatty acids like linoleic acid and arachidonic acid, hydroxy and dihydroxy octadecadienoic acid, lysophospholipids and sphingolipids can sensitize nociceptor neurons to alter pain sensitization either directly by activating nociceptors, ion channels and G-protein-coupled receptors or indirectly through inflammatory mediators and intracellular protein kinases [51,52,53]. The pain aggravation effect of arachidonic acid-derived prostaglandins is well documented, and these factors either act as intracellular pro-inflammatory mediators or directly incite peripheral sensitization [54].

Taken together, various cellular metabolites modulate vital biological pathways and contribute to inflammation, pain sensitization and synaptic plasticity through intricate and diverse mechanisms, highlighting a dynamic interplay between metabolic pathways and immune responses deriving cellular machinery in an altered functional way.

## 4. Oxidative Stress and Mitochondrial Dysfunction in the Pathogenesis of Orofacial Pain

The involvement of perturbed redox homeostasis owing to oxidative stress generated by reactive oxygen species (ROS) such as hydroxyl radical (OH^−^), hydrogen peroxide (H_2_O_2_) and superoxide anions (O_2_^−^) as well as reactive nitrogen species (RNS) such as nitric oxide (NO^−^) and its derivatives has been observed in orofacial pain. It can significantly impact the etiology and modulation of orofacial pain, and with the availability of enough literature, we can comprehend a mutually reciprocating rapport between pain and cellular redox status. It is established that ROS perturbations in sensory neurons influence pain intensity or hyperalgesia as well as neuronal plasticity by regulating the excitability of respective neurons [55].

Previous studies indicate that oxidative stress in the trigeminal nucleus is involved in orofacial pain in a rat model [55,56]. Clinical studies have established a direct correlation between oxidative stress and pain intensity in patients with TMJ disorders [57,58]. The evaluation of oxidative stress biomarkers such as 8-Hydroxydeoxyguanosine (8-OHdG), protein carbonylation (PC), malondialdehyde (MDA) or nuclear factor erythroid 2-related factor 2 (Nrf2) is also being reserved as a diagnostic tool for TMJ disorders [59,60,61]. There are reports suggesting immunomodulatory effects of oxidative stress generating differential pain phenotypes. ROS increment can lead to a pro-inflammatory response in our nervous system through factors such as IL-1β, COX-2, the mitogen-activated protein kinase (MAPK) pathway, toll-like receptors and TNF-α, thereby aggravating pain conditions [55].

As for the role of ROS in neurodegenerative diseases, ROS are involved in almost every pathway of neuropathic pain, which indicates their contribution to pain pathophysiology. On the other hand, it is also established that the redox status of the cell plays a decisive role in regulating mitochondrial function and remodeling, a phenomenon characterized by the differential distribution of mitochondria on the basis of their shape and size [62]. However, studies have not explored whether mitochondrial dysfunction has a role in orofacial pain. It has been reported that people with heritable mitochondrial diseases experience chronic pain, suggesting a direct correlation between chronic pain and mitochondrial disturbance [63]. Electron transport chain inhibitors affecting respiration and ATP production, antioxidants scavenging accumulated ROS or mitochondrial Ca^+2^ uptake regulation has shown inhibitory effects on nociceptive behavior in different pain models [64]. Persistent mitochondrial disturbance in the soma of sensory neurons further triggers pain signaling and causes a transition to chronic pain [7]. Accumulated ROS as well as mitochondrial dysfunction can also result in dysregulated autophagy, thereby aggravating pain conditions [6].

## 5. Epigenetic Regulation of Orofacial Pain

Epigenetics deals with DNA methylation and histone modification of our genetic system at the chromatin level to regulate gene expression without any change in the primary structure of DNA. These alterations are heritable and can elicit a distinct phenotype. It primarily navigates the accessibility of genetic sequence to be expressed in an altered way by modulating the binding of transcription factors or by chromatin remodeling affecting proximity to the DNA strand [65]. Altered pain behavior can be manifested by DNA methylation, histone modification such as acetylation, methylation, phosphorylation, ADP-ribosylation, and miRNA expression [66]. A genome-wide assessment of DNA methylation further validates the role of epigenetics in chronic pain [67]. Additionally, perturbed cellular oxidative machinery is accompanied by an imbalanced NAD^+^/NADH ratio that impacts the histone acetylation or deacetylation process, thereby sparking epigenetic modifications in pain signaling [8,39].

Histone modification, DNA methylation, and non-coding RNA modification are critical for the modulation of neuropathic pain [68]. There have been at least 358 genes, including voltage-gated sodium channel-type 1-alpha subunit (*SCN1A*), angiotensin I-converting enzyme 2 (*ACE2*), prostaglandin-endoperoxide synthase 1 (*PTGS1*) and amyloid-beta precursor protein (*APP*), identified to be associated with TMD pain [69]. In a rat TMJ osteoarthritis model, DNA methylation has been shown to contribute to inflammation, immune response, angiogenesis and ossification, directing the remodeling of respective joint structures [70]. *BDNF* is an important neurotrophin involved in neuronal plasticity and nociceptive excitability [71], and its gene can be modified at both the DNA and histone levels. DNA comprising exon III of the gene has been found to be methylated, whereas there is an increment in histone dimethylation of k4 and k9 and a decrement in k29 acetylation [72].

**Figure 2:**
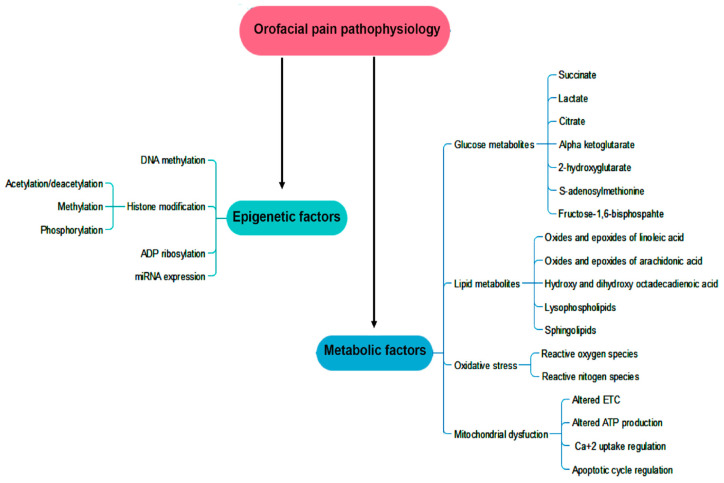
Orofacial pain pathophysiology is controlled by a multitude of factors encompassing metabolic and epigenetic elements. The metabolic factors include glucose and lipid metabolites along with reactive species regulating mitochondrial function, and the epigenetic mechanism is mainly composed of DNA methylation and histone modification.

Owing to the profound impact of histone deacetylation catalyzed by histone deacetylases (HDACs) on craniofacial pain intensity, HDAC inhibitors, such as HDAC5 inhibitor LMK253, have demonstrated promising therapeutic potential for such pain [73]. It has also been reported that persistent pain accompanies the epigenetic suppression of the *Gad2* gene, which encodes glutamic acid decarboxylase 65, which impairs GABAergic synaptic inhibition via HDAC-mediated histone hypoacetylation [74]. These observations highlight the impact of epigenetic regulation on pain mechanisms. Figure 2 depicts all the factors that contribute to pain pathophysiology.

In addition, the role of non-coding RNAs in pain signaling is also being investigated [75]. Non-coding RNAs comprise part of transcribed RNAs that do not code for proteins [76], but they exhibit regulatory effects on various signaling pathways, gene transcription and chromatin remodeling and can act as oncogenic and tumor-suppressive factors [77,78,79]. Also, their regulatory effects on the wiring and remodeling of neural circuits have been very well established [80]. Thus, many disease conditions have now been identified with dysregulated non-coding RNAs [81], indicating that they can serve as potential biomarkers for various clinical conditions including cancer [82,83]. Various studies have also shown that these non-coding RNAs play a crucial role in oral inflammation and lesion formation, suggesting their foothold in the signaling of oral pain pathways [75,84]. Clinically, microRNAs, among other non-coding RNAs, exhibit significant regulatory effects on several oral pathologies like pulpitis, periodontitis, oral lichen planus and gingival epithelium. Studies on various temporomandibular complications have also validated the role of dysregulated microRNAs in inflammation and inflammatory pain [75]. A study on injured lingual tissues has shown that the dysregulation of several microRNAs like miR-138, miR-138 and miR-29a can cause augmented pain behavior [85], whereas another study on saliva samples from burning mouth syndrome observed the upregulation of miR-1273h-5p/1273a/1304-3p/4449/1285-3p/6802-5p/1268a/1273d/1273f/423-5p and downregulation of miR-27b-3p/16-5p/186-5p/142-3p/141-3p/150-5p/374a-5p/93-5p/29c-3p/29a-3p/148a-3p/22-3p/27a-3p/424-5p/19b-3p/99a-5p/548d-3p/19a-3p, suggesting a role of these microRNAs in the development of neuropathic orofacial pain [86]. Additionally, in serum samples from patients with trigeminal neuralgia, dysregulation in the levels of microRNAs such as miR-132-3p, miR-146b-5p, miR-155-5p and miR-384 contributes to the development of pain [87]. The role of MRAK009713 in regulating the expression and function of the P2X3 receptor suggests its involvement in neuropathic pain [88]. It has been reported that the interactions among non-coding RNAs and mRNAs can cause pain augmentation through the regulation of pain-related genes and nociceptive signaling [89].

## 6. Primary Experimental or Clinical Data in Orofacial Pain Research

An experimental mechanistic insight into pain pathology provides significant primary data, which could serve as a basis for human clinical studies to pave the way for developing advanced therapeutics. Presently, clinical trials for the management of TMDs and trigeminal neuralgia involve a range of pharmacological treatments targeting various physiological pathways. A study on 60 patients experiencing sleep bruxism and muscle-related TMDs demonstrates the pain-alleviating effect of cannabidiol formulations [90]. These formulations target the endocannabinoid system, involving the regulation of lipid messengers on specific cannabinoid receptors both at peripheral and central levels, thus ameliorating inflammation and neuropathic orofacial pain [91]. Also, several clinical trials have studied formulations like gabapentin and botulinum toxins to explore their pain-alleviating potential [92,93,94,95,96]. In addition to drug-based treatment, current clinical paradigms have incorporated other therapies like physical therapy, cognitive behavior therapy, laser acupuncture therapy, transcranial magnetic stimulation and stem cell therapies to examine their advances in pain management [97,98,99,100,101,102,103,104,105,106,107]. Thus, strategic and evidential collaborations of the scientific community including scientific researchers and clinicians worldwide can benefit from the above explained multidimensional approaches inclusive of all immunomodulating, metabolic and epigenetic factors.

## 7. Roles of Psychosocial Factors and Systemic Health Conditions in Orofacial Pain

As the diagnosis of the origin of orofacial pain involves a thorough investigation into etiological factors along with odontogenic factors, it is now very well established that patients’ psychological status and systemic health conditions play conspicuous roles in the development and exacerbation of orofacial pain. Several studies have shown a positive correlation between pain associated with TMDs and depression, anxiety, stress or emotional distress. This correlation can be attributed to the altered neuroplasticity and disturbed neurobiological homeostasis involving neuroendocrine dysfunction and chronic inflammation during the development of orofacial pain [108,109,110,111,112,113,114,115,116]. A case study on patients with orofacial pain and early-onset diabetes has shown that the experience of nociceptive pain is exacerbated by diabetes, thereby validating a link between orofacial pain and systemic health conditions [117]. Another case study has demonstrated that trigeminal nociception, intraoral sensitization and structural nerve change are related to diabetic peripheral neuropathy [118]. Moreover, some investigations have indicated the fact that the risk of periodontitis is increased by type 1 and 2 diabetes [119,120,121,122]. In addition, coronary artery diseases or ischemic heart diseases can cause orofacial pain, which can be used as a diagnostic symptom for further cardiac malfunction [123,124,125].

## 8. Conclusions

**Figure 3:**
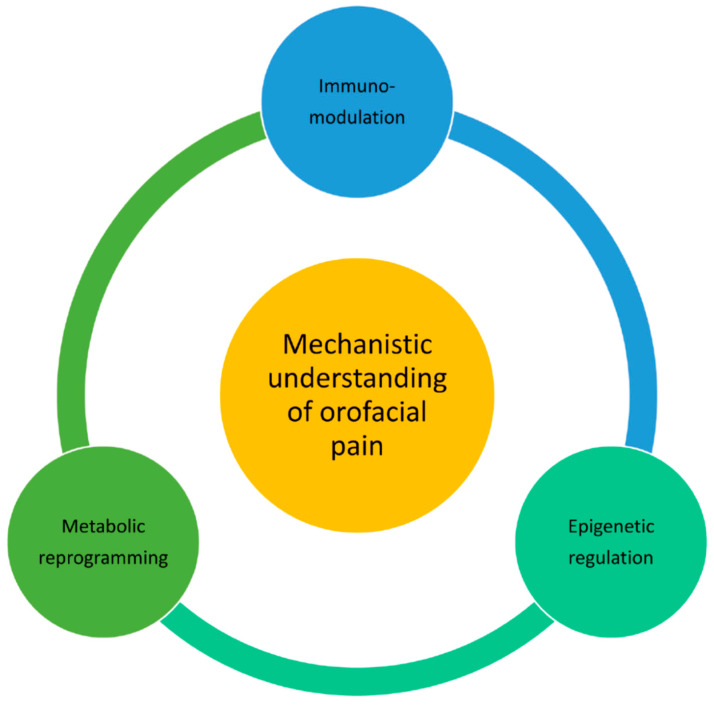
A holistic approach integrating immunomodulatory, metabolic and epigenetic regulation of pain pathways to fully elucidate mechanisms underlying pain pathophysiology.

A profound understanding of pain pathogenesis and mechanisms requires a comprehensive approach that encompasses the co-interaction of various immunomodulatory, metabolic and epigenetic factors (Figure 3). It is imperative to create a holistic treatment strategy targeting multimodal mechanisms behind the multitude of observed systemic and localized pain perceptions culminating in differential pain behaviors. The pathophysiology of orofacial pain involves an interplay of the immune and nervous systems under the direction of fluctuating oxidative metabolism and genetic regulation. Glucose and lipid metabolites modulate several critical physiological pathways with perturbed redox homeostasis. The generated ROS interfere with cellular machinery accompanied by further molecular perturbations along with epigenetic modulation. The link between oxidative stress and epigenetic modulation emphasizes the critical role of the metabolic factor in chronic orofacial pain. This comprehensive review bridges our understanding of orofacial pain pathology from the molecular level to the gene level; therefore, it provides a window for various researchers and clinicians to understand the mechanisms of orofacial pain.
